# Engineering archaeal membrane‐spanning lipid GDGT biosynthesis in bacteria: Implications for early life membrane transformations

**DOI:** 10.1002/mlf2.70001

**Published:** 2025-03-13

**Authors:** Huahui Chen, Fengfeng Zheng, Xi Feng, Zijing Huang, Wei Yang, Chuanlun Zhang, Wenbin Du, Kira S. Makarova, Eugene V. Koonin, Zhirui Zeng

**Affiliations:** ^1^ Department of Ocean Science and Engineering Southern University of Science and Technology Shenzhen China; ^2^ State Key Laboratory of Microbial Resources, Institute of Microbiology Chinese Academy of Sciences Beijing China; ^3^ National Center for Biotechnology Information, National Library of Medicine Bethesda Maryland USA

**Keywords:** archaeal lipid GDGT, cellular filamentation, eukaryogenesis, hybrid membrane, SOS response

## Abstract

Eukaryotes are hypothesized to be archaeal–bacterial chimeras. Given the different chemical structures of membrane phospholipids in archaea and bacteria, transformations of membranes during eukaryogenesis that led to the bacterial‐type membranes of eukaryotic cells remain a major conundrum. One of the possible intermediates of eukaryogenesis could involve an archaeal–bacterial hybrid membrane. So far, organisms with hybrid membranes have not been discovered, and experimentation on such membranes has been limited. To generate mixed membranes, we reconstructed the archaeal membrane lipid biosynthesis pathway in *Escherichia coli*, creating three strains that individually produced archaeal lipids ranging from simple, such as DGGGOH (digeranylgeranylglycerol) and archaeol, to complex, such as GDGT (glycerol dialkyl glycerol tetraether). The physiological responses became more pronounced as the hybrid membrane incorporated more complex archaeal membrane lipids. In particular, biosynthesis of GDGT induced a pronounced SOS response, accompanied by cellular filamentation, explosive cell lysis, and ATP accumulation. Thus, bacteria seem to be able to incorporate simple archaeal membrane lipids, such as DGGGOH and archaeol, without major fitness costs, compatible with the involvement of hybrid membranes at the early stages of cell evolution and in eukaryogenesis. By contrast, the acquisition of more complex, structurally diverse membrane lipids, such as GDGT, appears to be strongly deleterious to bacteria, suggesting that this type of lipid is an archaeal innovation.

## Impact statement

The formation of archaeal–bacterial hybrid membranes represents possible intermediates in early life evolution and eukaryogenesis. In this study, we synthesized archaeal membrane‐spanning lipid GDGT (glycerol dialkyl glycerol tetraether) in bacteria *Escherichia coli* to create such hybrid membranes and examined their physiological properties. Our results suggest that the formation of hybrid membranes containing simple archaeal‐type lipids could be a plausible intermediate stage at major evolutionary transitions, whereas more complex, membrane‐spanning lipid GDGT would have been deleterious and probably represent a later archaeal innovation, possibly an adaption to thermal habitats. These findings provide new insights and perspectives into the evolution of membranes at the earliest stages of cellular evolution as well as at eukaryogenesis.

## INTRODUCTION

The membrane lipids in bacteria, eukaryotes, and archaea exhibit major differences. In bacteria and eukaryotes, the principal membrane building blocks are diacylglycerol (DAG)‐based lipids composed of two fatty acid chains ester‐linked to glycerol‐3‐phosphate (G‐3‐P). In contrast, the archaeal membrane lipids are based on two isoprenoid chains ether‐linked to glycerol‐1‐phosphate (G‐1‐P)^1^. In addition to diether lipids (archaeol), a major component of the archaeal membrane is tetraether lipids (glycerol dialkyl glycerol tetraethers, GDGTs)^2^. Archaeol forms bilayers structurally similar to bacterial and eukaryotic membranes whereas GDGTs are membrane‐spanning lipids that form monolayers^3^. The formation of GDGTs‐based monolayer membrane enhances membrane stability and confers tolerance to extreme conditions such as high temperature and acidity^4^, ^5^, ^6^, ^7^, ^8^. Notably, some bacteria also produce membrane‐spanning lipids, structurally analogous to archaeal GDGTs, known as branched GDGTs (brGDGTs)^9^, ^10^, ^11^, ^12^. However, the biosynthesis pathways and biological functions of brGDGTs remain largely unknown.

The evolution of these two types of membrane lipids at the early stages of cellular evolution remains an open problem^13^, ^14^, ^15^, ^16^. Apparently, the divergence of bacteria and archaea from the last universal cellular ancestor (LUCA) resulted in a “lipid divide” between bacteria and archaea, suggesting that the LUCA might have had a mixed hybrid membrane^17^, ^18^. At a later stage of evolution, eukaryogenesis might involve the emergence of an archaeal–bacterial chimera via endosymbiosis. One scenario of eukaryogenesis includes the capture of an alpha‐proteobacterial endosymbiont, the ancestor of the mitochondria, by an Asgard archaeal host, which would require a transition from the archaeal membrane to the bacterial‐type membrane found in all eukaryotes, possibly, via a mixed membrane intermediate^19^, ^20^, ^21^, ^22^. An alternative scenario postulates a bacterial host (possibly a delta‐proteobacterium) that hosted first an Asgard archaeal and, subsequently, an alpha‐proteobacterial endosymbiont^23^, ^24^, ^25^. Under this model, the continuity of the bacterial membrane would be preserved throughout the eukaryogenesis, but nevertheless, an evolutionary intermediate would possess two distinct systems of phospholipid biosynthesis, with a potential for the formation of a mixed membrane. Thus, an archaeal–bacterial hybrid membrane might have been involved in two landmark events in the early evolution of life: the emergence of the LUCA and the last eukaryotic common ancestor (LECA).

To date, no organisms with archaeal–bacterial hybrid membranes have been isolated or cultured. As an alternative approach to exploring the physiological features of hybrid membranes, researchers have turned to engineering organisms producing both types of phospholipids. Lai et al.^26^ first successfully reconstructed of archaeal unsaturated diether lipid (DGGGOH) biosynthesis in *Escherichia coli*. Subsequent advancements have enabled the synthesis of saturated diether lipid (archaeol), albeit in relatively small amounts^27^, ^28^. The efforts in this direction have significantly elevated the production of archaeal unsaturated diether lipids in *E. coli* membrane, allowing for the determination of the hybrid membrane stability^29^, ^30^. In addition to using *E. coli* as a host, yeast *Saccharomyces cerevisiae* was also engineered to synthesize archaeal unsaturated diether lipids that formed a hybrid membrane in a eukaryotic host^31^.

However, in all previous studies, only hybrid membranes containing archaeal diether lipids, but not membrane‐spanning GDGT lipids, were produced in engineered bacteria. The GDGTs are the major membrane lipids in most archaea, including cultivated representatives of *Asgardarchaeota*, the phylum that is thought to include the archaeal ancestor of eukaryotes^32^. Recent studies have revealed the biosynthesis pathway for GDGTs, including GDGTs synthase (Tes)^33^, ^34^, GDGTs calditol headgroup synthase (Cds)^35^, and GDGTs ring synthase (Grs)^36^, enabling the engineering of GDGTs synthesis in model organisms.

In this work, we engineered both archaeol and GDGT synthesis in *E. coli* and demonstrated hybrid membrane formation. We found that, unlike mixed membranes containing diether lipids, incorporation of GDGT in the bacterial membrane induced a pronounced physiological response, with implications for the early evolution of membranes.

## RESULTS

### Biosynthesis of archaeal lipids, archaeol, and GDGT in *E. coli* DH10B

**Figure 1:**
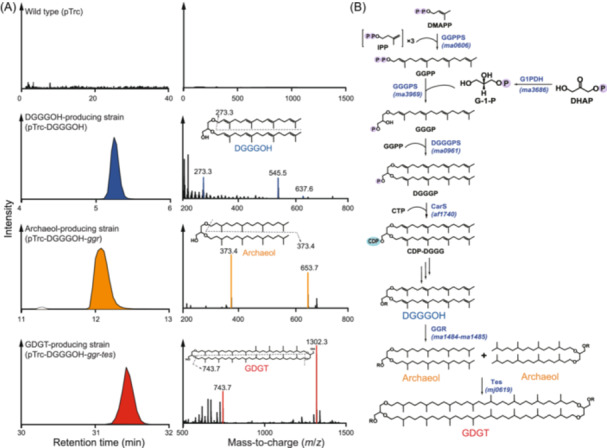
Biosynthesis of archaeal membrane lipids DGGGOH, archaeol, and GDGT in *Escherichia coli*. (A) Identification of archaeal lipids in *E. coli* strains. The left column is the liquid chromatography‐mass spectrometry (LC‐MS) extracted ion chromatograms (EICs) of lipid extracts from various *E. coli* transformants. The right column is the tandem mass spectrometry (MS/MS) spectra of the corresponding compounds from the left column. The intact polar lipids (IPLs) extracted from DGGGOH‐producing strain and the core lipids (CLs) extracted from archaeol‐ and GDGT‐producing strains are analyzed. (B) The archaeal lipid biosynthetic pathway reconstructed in *E. coli*. CarS, CDP‐archaeol synthase; DGGGOH, digeranylgeranylglycerol; DGGGPS, digeranylgeranylglyceryl phosphate synthase; G1PDH, G‐1‐P dehydrogenase; GGGPS, geranylgeranylglycerol phosphate synthase; GGPPS, geranylgeranyl pyrophosphate synthase; GGR, geranylgeranyl reductase; Tes, tetraether synthase.

To express archaeal membrane‐spanning lipid GDGT in *E. coli*, we cloned 8 archaeal membrane lipids biosynthetic genes into the plasmid pTrc (Figure S1). We first constructed the pTrc‐DGGGOH plasmid containing five genes encoding geranylgeranyl pyrophosphate synthase (GGPPS, *ma0606*), digeranylgeranylglyceryl phosphate synthase (DGGGPS, *ma0961*), geranylgeranylglyceryl phosphate synthase (GGGPS, *ma3969*), glycerol‐1‐phosphate dehydrogenase (G1PDH, *ma3686*), and CDP‐archaeol synthase (CarS, *af1740*), enabling the production of the unsaturated diether lipid DGGGOH (digeranylgeranylglycerol) (Figure 1A,B), as described previously^27^. In particular, the function of CarS is to modify the lipid headgroups, facilitating the fusion of archaeal lipids with *E. coli* cell membranes^37^. Next, we added the genes encoding geranylgeranyl reductase with its ferredoxin (GGR, *ma1484‐1485*) to generate the plasmid pTrc‐DGGGOH‐*ggr* for the synthesis of saturated diether lipid archaeol (Figure 1A,B). Archaeol was primarily synthesized under anaerobic conditions, with limited production in aerobic conditions, suggesting that the function of GGR requires a reducing intercellular environment, consistent with a previous report^38^. Finally, we added the *tes* gene encoding tetraether synthase (Tes, *mj0619*), resulting in the plasmid pTrc‐DGGGOH*‐ggr‐tes* which drove the synthesis of GDGT in *E. coli* under anaerobic conditions (Figure 1A,B). In addition to GDGT, the products of the *tes* gene included a small amount of macrocyclic archaeol and GTGT (glycerol trialkyl glycerol tetraether)^33^ (Figure S2).

Three auxiliary plasmids were used to promote the production of archaeal lipids in *E. coli*. The plasmid pJBEI‐2997 enhanced the mevalonate pathway to produce more isoprenoid units in *E. coli* ^39^, ^40^, the plasmid pBAD42‐BtuCEDFB enhanced the solubility of radical S‐adenosylmethionine (SAM) superfamily proteins in *E. coli* ^41^, and the plasmid pDB1281 facilitated the maturation of iron‐sulfur clusters for radical SAM proteins^42^, ^43^, ^44^. The co‐transformation of these three auxiliary plasmids with the pTrc plasmid led to substantial production of mature archaeal lipids (archaeol and GDGT) in anaerobic cultures. The DGGGOH‐producing strain synthesized approximately 3% DGGGOH of the total bacterial membrane lipids (Figure S3A). The archaeol‐producing strain synthesized about 0.6% DGGGOH and 3.8% archaeol of the total lipids (Figure S3B). The GDGT‐producing strain synthesized about 3.6% DGGGOH, 3.1% archaeol, and 0.4% GDGT of the total bacterial membrane lipids (Figure S4). DGGGOH lipids with PG (phosphatidylglycerol) head group were detected in the DGGGOH‐producing strain, consistent with previous reports^29^, ^30^. Archaeol with PE (phosphatidylethanolamine) and PG head group was detected in the archaeol‐producing strain. Due to the low abundance of GDGT extracted by the Bligh‐Dyer method, intact polar GDGT with any head group was not detected in the GDGT‐producing strain.

To assess whether the synthesized archaeal lipids were incorporated into the *E. coli* cell membrane, we separated the membrane fractions and the cytoplasmic fraction by ultracentrifugation (240,000*g*) as reported previously^45^. The lipid analysis indicated that the synthesized archaeal lipids predominantly localized to the membrane fraction, which exhibited nearly twice the archaeal lipid content compared to the cytosolic fraction (*t*‐test, *p* < 0.05, Figure S5). Thus, the synthesized archaeal lipids were incorporated into the cell membrane, forming an archaeal–bacterial hybrid membrane in *E. coli*.

### Hybrid membrane induces cellular filamentation and causes explosive cell lysis

**Figure 2:**
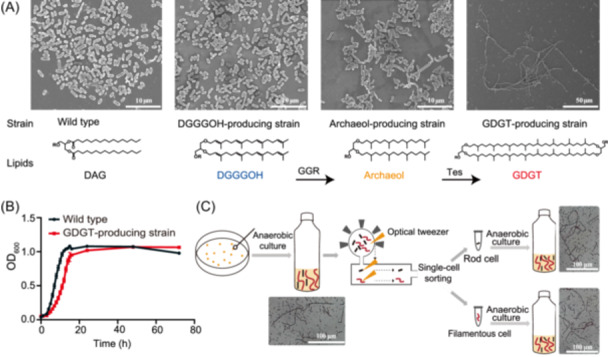
GDGT synthesis induces cellular filamentation in *E. coli*. (A) More complex archaeal lipid synthesis resulting in significantly longer filamentous cells. Cell morphology of various *E. coli* transformants with corresponding archaeal lipid synthesis was observed by the scanning electron microscope (SEM). (B) GDGT‐producing strain exhibiting slightly slower growth compared to the wild type during the early logarithmic phase. The growth curves of the GDGT‐producing strain and the wild‐type strain were determined by optical density measurement under anaerobic conditions at 37°C. However, both strains ultimately achieve similar cell densities by the stationary phase. (C) The reversible changes in cellular morphology of GDGT‐producing strain. The single‐cell sorting system was used to isolate rod and filamentous cells from the GDGT‐producing strain for subsequent cultures.

The pronounced phenotype observed in *E. coli*‐producing archaeal lipids was cell filamentation. The length of the filamentous cells depended on the type of archaeal lipids synthesized, with more complex lipid synthesis resulting in much longer filamentous cells (Figure 2A). The wild‐type *E. coli* (pTrc) and the DGGGOH‐producing strain (pTrc‐DGGGOH) typically had lengths of 1–2 μm, whereas some archaeol‐producing cells (pTrc‐DGGGOH‐*ggr*) reached about 10 μm in length. Strikingly, the GDGT‐producing cells (pTrc‐DGGGOH‐*ggr*‐*tes*) elongated up to 250 μm although the cell population was highly heterogeneous, with some cells maintaining a regular rod shape similar to the wild type (Figure 2A). The growth of the GDGT‐producing strain was slightly slower than the growth of the wild type during the early log phase, but it eventually reached a similar cell density at the stationary phase (Figure 2B). The culture of DGGGOH‐ and archaeol‐producing strains, for unknown reasons, induced black precipitation in an anaerobic medium (Figure S6), making optical density measurements impractical for monitoring growth.

To explore the morphological diversity in the GDGT‐producing strain, we isolated the rod and filamentous cells by single‐cell sorting using an optical tweezer‐assisted pool‐screening system. Despite culturing these two cell types separately, they both reverted to the mixture of rod and filamentous morphology resembling their parental culture (Figure 2C), indicating that the cell shape change is reversible, such that rod‐shaped cells can undergo elongation, whereas filamentous cells can resume a rod shape.

**Figure 3:**
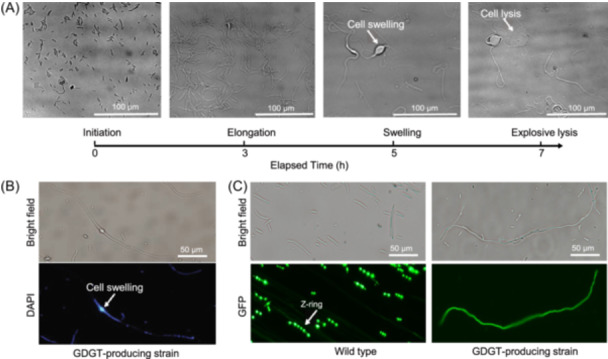
Explosive cell lysis and cell division inhibition in the GDGT‐producing strain. (A) The elongation process of the GDGT‐producing strain ending with explosive cell lysis illustrated by the live‐cell time‐lapse microscopy imaging. (B) The cell swelling site containing high DNA content shown by DNA staining with DAPI. (C) The Z‐rings formation inhibited in the GDGT‐producing strain. The GFP‐fused FtsZ proteins aggregated at the middle of the cell, forming a Z‐ring to facilitate cell division in the wild‐type strain, but they were distributed along the whole filamentous cell without Z‐ring formation in the GDGT‐producing strain.

To observe the process of cell elongation, we performed live‐cell time‐lapse microscopy analysis during the cell growth. The imaging captured the initiation of the elongation process originating from a rod‐shaped cell (Figure 3A; Movie S1). Notably, we observed explosive cell lysis in certain filamentous cells during the late elongation stage (Figure 3A; Movie S1). After the lysis, the filamentous cells ceased growth, indicating cell death or dormancy. To identify the cause of explosive cell lysis, we stained DNA with DAPI (4′, 6‐diamidino‐2‐phenylindole) and found that the swelling site formed on filamentous cells contained large amounts of DNA (Figure 3B), suggesting that rapid DNA synthesis and accumulation induced cell lysis. Moreover, the DNA staining images also revealed the presence of multiple, non‐uniformly distributed genomes in filamentous cells (Figure 3B).

The abnormal filamentation in *E. coli* is usually attributed to inhibition of cell division. The FtsZ protein plays a key role in cell division by forming the Z‐ring^46^. We tracked the distribution of FtsZ protein within the cells by fusing it with green fluorescent protein (GFP). In wild‐type *E. coli*, FtsZ was observed to aggregate in the middle, forming the typical Z‐ring^47^ (Figure 3C). By contrast, in the GDGT‐producing strain, FtsZ was widely distributed along the entire filamentous cell (Figure 3C), indicating the inhibition of Z‐ring formation and the consequent blockage of cell division.

The phenotype of both the DGGGOH‐producing and the archaeol‐producing strains remained stable throughout serial passages. By contrast, the GDGT‐producing strain exhibited a loss of traits after two to three passages. Plasmid sequence analysis showed that the trait loss in the GDGT‐producing strain was associated either with mutations in the *tes* gene alone or with additional mutations in the promoter of the pTrc plasmid, resulting in the cessation of GDGT synthesis in *E. coli* (Figure S7). These findings indicate that the synthesis of GDGT, but not DGGGOH and archaeol, imposes substantial physiological stress on *E. coli*, resulting in selection pressure for phenotype‐reversing mutations.

### GDGT biosynthesis triggers SOS response and ATP accumulation in *E. coli*

**Figure 4:**
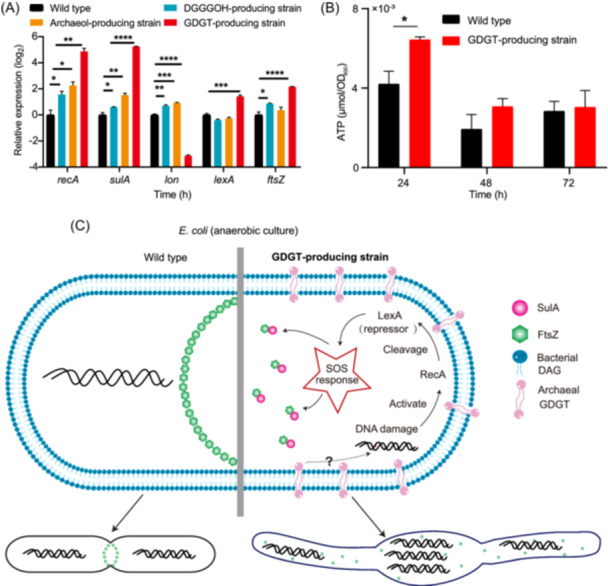
Biosynthesis of GDGT in bacteria triggers SOS response and ATP accumulation. (A) The relative expression levels of the SOS response‐related genes in the DGGGOH‐, archaeol‐ and GDGT‐producing strains compared to the wild‐type strain revealed by qRT‐PCR analysis. The housekeeping gene *rssA* was used as the internal reference. (B) The intercellular ATP concentrations of the GDGT‐producing strain and the wild‐type strain at different growth time points. Data were calculated from three biological replicates, and the error bars stand for standard deviation. *p < 0.05; **p < 0.01; ***p < 0.001; ****p < 0.0001. (C) The schematic diagram of the proposed mechanism for induction of SOS response by GDGT synthesis in *E. coli*.

Cellular filamentation in *E. coli* is associated with SOS response, which is mediated by the RecA‐LexA system and is usually triggered by DNA damage^48^, ^49^. We applied quantitative reverse‐transcription PCR (qRT‐PCR) to monitor the expression of SOS response‐related genes (*recA*, *lexA*, *sulA*, *lon*, and *ftsZ*)^50^ in the wild type, DGGGOH‐, archaeol‐, and GDGT‐producing strains. The expression profiles of these genes in the GDGT‐producing strain were consistent with the SOS response (Figure 4A). Specifically, the significant upregulation of *recA* (29‐fold, *p* < 0.01) (Figure 4A) suggests that increased production of RecA enhanced the cleavage of the SOS repressor LexA^51^ and allowed transcription of SOS‐related genes. Notably, the *sulA* gene exhibited an even more pronounced upregulation than *recA*, 38‐fold (*p* < 0.0001) in the GDGT‐producing strain. The SulA protein, the expression of which is triggered by SOS response^52^, blocks FtsZ polymerization, preventing the Z‐ring formation and thus inhibiting cell division^53^. Furthermore, the *lon* gene encoding the Lon protease responsible for SulA degradation^54^, ^55^ was downregulated approximately 9‐fold (*p* < 0.0001). For the DGGGOH‐ and archaeol‐producing strains, the expression changes of SOS response genes were mild and, in some cases, showed no significant difference compared to the wild‐type strain (Figure 4A). Taken together, these findings show that biosynthesis of archaeal lipid GDGT in *E. coli* induced a pronounced SOS response, leading to cellular filamentation and explosive lysis of some cells (Figure 4C), whereas the production of DGGGOH and archaeol induced weak or no SOS response.

The SOS response is known to change the energy state of bacterial cells by increasing the ATP concentration approximately 2‐fold^56^. To monitor the energy state of the hybrid membrane cells, we used the luminescence assay to measure the amount of ATP. During the first 24 h of incubation, the intracellular ATP concentration in the GDGT‐producing strain was 1.5–2‐fold higher than that in the wild‐type strain with statistical significance (*t*‐test, *p* = 0.03) (Figure 4B). By contrast, at the 48 and 72 h incubation, the ATP concentration in the GDGT‐producing strain did not show a significant difference from the wild type (Figure 4B).

### Representation of the GDGTs synthetic gene *tes* in Asgard lineages

**Figure 5:**
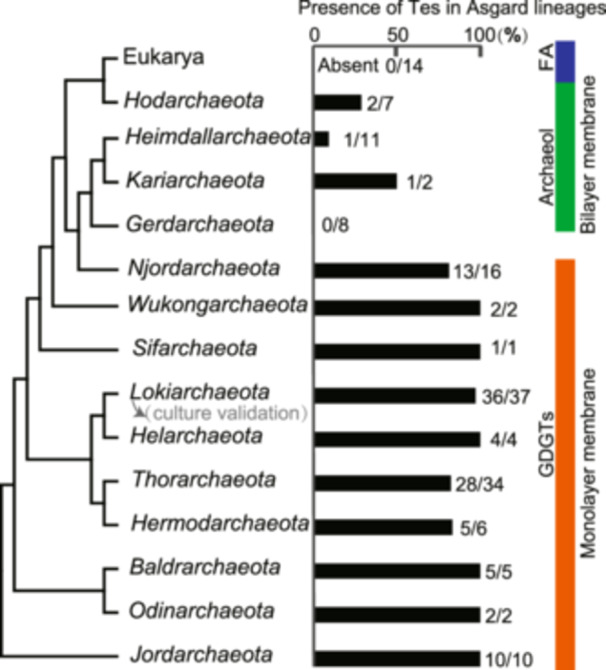
Phylogenetic tree of Asgard lineages and their distribution of the *tes* gene. The tree topology was modified from Eme et al.^57^ and the bars on the right show the fraction of the examined Asgard archaeal genomes in each lineage that contained the *tes* gene responsible for GDGTs biosynthesis. FA, fatty acids.

We analyzed 145 genomes of *Asgardarchaeota* covering all major Asgard lineages^57^, ^58^ and protein sequences of lipid biosynthesis enzymes were retrieved from the database of the Asgard clusters of orthologous genes (asCOGs)^58^. Most of the Asgard lineages were found to encompass the *tes* gene involved in GDGTs biosynthesis (Figure 5; Table S1), in particular, the class of *Lokiarchaeia* for which the presence of GDGTs as the major membrane lipids has been validated biochemically^32^. Notably, however, most of the members of the classes *Hodarchaeia* and *Heimdallarchaeia*, the closest relatives of eukaryotes^57^, ^58^, lacked the *tes* gene, most likely, having lost it at early stages of their evolution. Thus, these organisms are predicted to form archaeol‐based bilayer membranes structurally similar to the fatty acids‐based bilayer membranes of bacteria and eukarya.

## DISCUSSION

Here, we advance the understanding of the biological impact of mixing bacterial and archaeal lipids in a membrane by revealing differential effects depending on the complexity of the lipid molecules. Simple diether archaeal lipids, DGGGOH and archaeol, when synthesized in *E. coli*, integrated into the bacterial membrane but had no to limited effect on the bacterial cell shape and division. In stark contrast, production of the complex, membrane‐spanning tetraether GDGT caused severe disruption of cell division accompanied by cell filamentation and lysis of some of the cells, which was apparently mediated via SOS response.

The SOS response is typically triggered by DNA damage^49^, ^59^. The connection between membrane distortion that is apparently caused by the incorporation of GDGT into the bacterial membrane and DNA damage remains to be fully investigated. The SOS response has been reported to be triggered by defective cell wall synthesis induced by β‐lactam antibiotics^60^. In this case, the proposed mechanism involves the generation of toxic reactive oxygen species (ROS), which causes DNA damage and subsequently activates SOS response^61^. However, in this work, *E. coli* was cultured anaerobically to induce GDGT formation, making the involvement of ROS unlikely^62^, ^63^. Nevertheless, even under anaerobic conditions, antibiotics could still induce SOS response in *E. coli* by generating other reactive electrophilic species that damage the cell membrane and DNA^64^. Membrane distortion caused by GDGT in *E. coli* might also induce reactive electrophilic species, resulting in DNA damage and triggering an SOS response (Figure 4C). The details of this process remain to be elucidated.

The incorporation of archaeol, which forms a bilayer membrane, only resulted in a minor phenotype in *E. coli* (Figure 2A). In contrast, the biosynthesis of GDGT, forming a monolayer, induced pronounced SOS response, cell filamentation, and lysis. Apparently, the conversion from a bilayer to a monolayer exerts a dramatic effect on bacterial cell physiology, which is consistent with the observations from in vitro liposome assays^65^, ^66^, ^67^, ^68^ and model simulations^69^, ^70^, ^71^, ^72^.

Our findings show that bacterial DAG backbone lipids are compatible with archaeal diether lipids, such DGGGOH^29^ and archaeol, without inducing major physiological stress in cells with a mixed membrane. Thus, at the early stages of evolution, cellular life forms, including the LUCA, might have had mixed membranes consisting of comparatively simple lipids, such as DAG‐based diester lipids intermixed with archaeol‐type diether lipids. Under this scenario, subsequent to the divergence of archaea and bacteria from the common ancestor, membrane lipids differentially homogenized in the two lineages, and biosynthesis of complex, membrane‐spanning lipids such as GDGT evolved in archaea, likely as an adaptation to life at high temperatures.

Regardless of the specific scenario of endosymbiosis, hybrid membranes likely also played a role in eukaryogenesis. Most of the extant archaea, in particular, members of the phylum *Asgardarchaeota*, possess genes involved in GDGT synthesis^33^ and produce GDGTs^32^ as their membrane lipids (Figure 5; Table S1). Notably, however, the classes within *Asgardarchaeota* that appear to be the closest known relatives of eukaryotes, *Heimdallarchaeia* and *Hodarchaeia* ^22^, ^57^, are mesophiles, and most of their genomes lack the *tes* gene responsible for GDGT synthesis (Figure 5; Table S1). Thus, these extant archaea, as well as the archaeal ancestor of eukaryotes, can be inferred to possess simple, bilayer membranes, which would facilitate endosymbiosis with the formation of a mixed membrane and subsequent evolution of eukaryotic cells.

## MATERIALS AND METHODS

### Microbial strains, media, and growth conditions

*E. coli* DH10B (Table S2) and its transformants were grown in Lysogeny Broth (LB) at 37°C with shaking at 200 rpm and supplemented with 70 μg/ml Carbenicillin, 50 μg/ml Kanamycin, 25 μg/ml Chloramphenicol, 50 μg/ml Spectinomycin and 50 μg/ml Gentamicin when needed, and all antibiotics came from Aladdin company in China. For growth on solid medium, LB medium was solidified with 1.5% (w/v) agar.

For archaeal lipids synthesis, *E. coli* transformants were grown under anaerobic conditions at 37°C in 100 ml of TM medium supplemented with an antibiotic to induce recombinant protein expression for producing archaeal lipids. TM medium^73^ was prepared by autoclaving a solution based on yeast extract (24 g/l), tryptone (20 g/l), glycerol (4 ml/l), with resazurin (1 mg/l) and by adding sterile 100 mM 3‐(Nmorpholino) propanesulfonic acid (MOPS, pH 7.8), 2 mM ammonium ferric citrate, 0.5% (w/v) glucose, 5 mM Cys‐HCl, and 10 mM fumarate. The wild type, DGGGOH‐, archaeol‐ and GDGT‐producing strains were induced with 0.2% l‐arabrose (w/v) and 0.5 mM IPTG for 72 h, to induce proteins overexpression.

### Molecular cloning

All plasmids and primers used in this study are listed in Tables S3 and S4. Primers were synthesized from Beijing Tsingke Biotech Company. Following the manufacturer's instructions, PCR amplification was conducted with either Rapid Taq Master Mix (Vazyme) or Phusion high‐fidelity DNA polymerase (New England Biolabs). For the extraction of *E. coli* plasmid DNA, the FastPure Plasmid Mini Kit (Vazyme) was employed. DNA fragments were purified using the FastPure Gel DNA Extraction Mini Kit (Vazyme). The ClonExpress MultS One Step Cloning Kit (Vazyme) was used to construct plasmids via stepwise Gibson assembly.

The plasmid pTrc^74^ was used to express archaeal ether lipids biosynthetic genes. The DNA fragments of G‐1‐P dehydrogenase (G1PDH, *ma3686*), GGPP synthase (GGPPS, *ma0606*), GGGP synthase (GGGPS, *ma3969*), and DGGGP synthase (DGGGPS, *ma0961*) were amplified from the genomic DNA of *Methanosarcina acetivorans* C2A. Additionally, the DNA fragments of CDP‐archaeol synthase (CarS, *af1740*) from *Archaeoglobus fulgidus* was codon optimized and synthesized by GENEWIZ Company. These fragments were then inserted into *Nco*I and *Xba*I sites of the plasmid pTrc using a stepwise Gibson assembly method to yield the DGGGOH‐producing plasmid pTrc‐DGGGOH. The *ggr* fragment (including *ma1484* and ferrodoxin *ma1485*) was amplified from *M. acetivorans* C2A genomic DNA by PCR and inserted into the *Xba*I sites of plasmid pTrc‐DGGGOH to yield the archaeol‐producing plasmid pTrc‐DGGGOH‐*ggr*. The codon‐optimized gene *tes* (*mj0619*) from *Methanocaldococcus jannaschii*, responsible for GDGT synthesis, was synthesized by GENEWIZ Company and cloned into the *Sal*I site of plasmid pTrc‐DGGGOH‐*ggr* to yield GDGT‐producing plasmid pTrc‐DGGGOH‐*ggr*‐*tes*. Plasmids pSRK^75^ was used as the basis to construct plasmids for controlled expression of the *ftsZ* fused with superfolder green fluorescent protein gene (*sfGFP*)^76^.

The wild‐type *ftsZ* gene was amplified from the genome DNA of the bacterium *E. coli* DH10B. Then, *ftsZ* and *sfGFP* fragments were inserted into the *Nde*I and *Hin*dIII sites of pSRK to create *ftsZ*‐*sfGFP* fusions. The expected DNA sequences of all cloned PCR products in this study were verified by Sanger sequencing at Beijing Tsingke Biotech Company.

### Transformation of plasmids into host cells

Electrocompetent *E. coli* DH10B cells were prepared following the protocol described by William et al.^77^. To elevate archaeal lipid production in *E. coli*, the plasmids pJBEI2997^39^ (producing isopentenyl diphosphate), pDB1281^42^, ^43^, ^44^ (producing iron‐sulfur clusters), and pBAD42‐BtuCEDFB^41^ (enhancing the solubilization of radical SAM proteins in *E. coli*) were co‐transformed into the *E. coli* cell together with pTrc plasmid. One microgram of plasmid was electroporated into 100 μl electrocompetent cells using a Micro‐Pulser Electroporator (Bio‐Rad) with 1.8 kV, 25 μF, 600 Ω in a 0.1 cm cuvette. Cells were recovered in LB media without antibiotic pressure at 37°C for 45 min and then sprayed on 1.5% agar LB plates with the needed antibiotic for 16–20 h at 37°C. Colonies appearing on selection plates were verified by colony PCR. Positive colonies were subsequently purified by streaking and verified by sequencing analysis.

### Lipid extraction and analyses

For core lipid (CL) and intact polar lipid (IPL) analyses, cultures of *E. coli* DH10B transformants (100 ml) were incubated to stationary phase around 72 h at 37°C, harvested by centrifugation at 10,000*g* for 10 min. A quantity of 100 ml of the cultures were split into two portions: one portion was utilized for CL extraction, while the other was used for IPL extraction. For CL extraction, cell pellets were subjected to acid hydrolysis following the method mentioned in Zeng et al.^33^ with some modifications. Briefly, 20 μl C_46_‐GTGT (concentration of 1.189 ng/μl) internal standard^78^ was added, and cell pellets were hydrolyzed with 5–10 ml solvent of methanol (MeOH): hydrochloric acid (HCl) = 9:1 (v:v) at 70°C for 8 h. After the reaction, 10 ml ultrapure water and 10 ml dichloromethane (DCM) were added to the solvent mixtures and vortexed for 1 min. Following a 5 min centrifugation at 2800*g* to effect separation of the aqueous and organic phases. The bottom organic phase, consisting of DCM, was carefully transferred to a fresh glass collection vial, and the residual aqueous phase underwent two additional extractions using freshly prepared DCM. Subsequently, all DCM fractions were pooled, passed through a 0.22 μm polytetrafluoroethylene (PTFE) filter to remove particulates, and then dried under a gentle stream of nitrogen gas.

The IPL extraction was extracted using the modified Bligh‐Dyer method^79^. Briefly, a subset of the cell pellets was extracted via sonication in a 9.5 ml mixture of MeOH: DCM: Nanopure water = 2:1:0.8 (v:v:v) for 30 min and added 10 ml Nanopure water and 10 ml DCM for phase separation by centrifugation and the DCM layer was collected. The residual liquid underwent two additional extractions with freshly prepared DCM. The collected organic phases were then filtered, dried using a stream of nitrogen gas as previously described, and stored at –80°C before further analysis.

The lipid analysis was performed using ultra‐performance liquid chromatography‐ion mobility‐quadrupole time‐of‐flight mass spectrometry (UPLC‐IM‐qTOF) at the Shenzhen Key Laboratory of Marine Archaea Geo‐Omics, Southern University of Science and Technology. A C18 EXCEL column (2.1 × 150 mm, 2 μm; ACE) was used to separate compounds with a flow rate of 0.3 ml/min maintained at 55°C. The elution gradient was set following the method described by Chen et al.^11^. Solvent A was MeOH (Optima™ LC/MS Grade, Fisher Chemical), and solvent B (Optima™ LC/MS Grade, Fisher Chemical) was isopropanol, both with 0.1% NH_3_OH (25%–30% NH_3_ basis; Sigma‐Aldrich) and 0.04% formic acid (>99.0%, Optima™ LC/MS Grade, Fisher Chemical). A quantity of 10 μl of the sample was injected and eluted in the following gradient: 100% A was maintained for the first 5 min, the gradient of solvent B linearly increased to 24% at 10 min, 60% at 36 min, 90% at 38 min and maintained for 7 min, then switch to 100% A at 45.1 min and re‐equilibration for 10 min. MS settings were identical to Chen et al.^11^: capillary voltage was set at 2.5 kV, source temperature at 120°C, sampling cone at 45, source offset at 80, desolvation gas flow at 800 l/h at 350°C, cone gas flow at 50 l/h, and nebulizer gas flow at 6.5 bar. The TOF analyzer was operated in the Resolution Mode, and the mass acquisition mode was FAST‐DDA with a mass range for MS^1^ *m*/*z* 100–2000 and MS^2^ *m*/*z* 50–2000 with the same scan time of 0.2 s. The top 5 ions within an intensity threshold >20,000 counts were fragmented using the transfer cell via collision‐induced dissolution (CID) to generate the MS^2^ spectra. The collision energy was ramped from 10 to 55 V for low mass and 15–65 V for high mass, respectively. An inclusion ion list of target GDGT compounds was used, and a real‐time dynamic exclusion of masses, with a duration of 5 s for both acquisition and exclusion, was activated to obtain MS^2^ spectra for ions of lower intensity. A solvent of sodium iodide (*m*/*z* 50–2000; residual mass error < 0.5 ppm) was used to calibrate the MS at the beginning of the experiments, and a leucine enkephalin solution was employed to perform real‐time mass calibration (scan time 0.2 s, interval 20 s) during the run.

The raw data were converted into mzML format and processed using the MS‐DIAL v4.9 software for lipidomic analysis^80^. Peak detection was performed with a minimum peak height of 1000 amplitude and mass slice width of 0.1 Da. The detected peaks were aligned with a retention time tolerance of 0.1 min and an MS^1^ tolerance of 0.05 Da. The adduct ions of [M+H]^+^ and [M+NH_4_]^+^ were considered and the mass features were annotated with a combination of the Lipidblast lipidomic library (http://prime.psc.riken.jp/compms/msdial/main.html) and in‐house archaeal lipid library^81^. The accurate mass tolerance for MS^1^ and MS^2^ was 0.01 and 0.05 Da, respectively. The identification score threshold was set to 70%, and the results were manually confirmed that only compounds with mass error <10 ppm remained. At last, we identified 88 compounds of bacterial phospholipids and six compounds of archaeal phospholipids to quantify the proportion of archaeal lipids synthesized to cell membrane lipids in *E. coli*.

### Separation of membrane and cytoplasmic fractions

The membrane and cytoplasmic fractions were separated following the protocol described by Jain et al.^45^. Cultures of *E. coli* DH10B transformants (100 ml) were incubated to stationary phase around 72 h and harvested by centrifugation at 10,000*g* for 10 min, then resuspended pellets in 10 ml lysis buffer (50 mM Tris‐HCl pH 7.5, 300 mM NaCl). The suspension was disrupted by sonication for 10 min (200 W, 3 s on, 3 s off) and centrifuged at low speed (4000*g*) for 30 min to remove unbroken cells. Then, the suspension was centrifugated with high‐speed centrifugation at 240,000*g* for 1 h to separate membrane fraction (in pellet) and cytoplasmic fraction (in suspension). Each fraction was collected, and lipid extraction was performed with acid hydrolysis.

### Scanning electron microscopy (SEM) imaging

Bacterial cells were prepared for SEM following the methods in Dong et al.^82^. The cultures were collected at the late logarithmic growth phase about 24 h under anaerobic conditions. Fresh‐grown *E. coli* cells were washed three times and subsequently resuspended in a phosphate‐buffered saline (PBS) buffer solution (0.1 M, pH 7.0). After that, cells were fixed overnight at 4°C using a 2.5% glutaraldehyde solution. Samples at the volume of 10 μl for each were loaded on a poly‐l‐lysine‐coated slide and air‐dried. Samples were dehydrated in a series of ethanol as follows: 30%, 50%, 70%, 90%, and 100% for 10 min each. Following the dehydration process, the samples were then affixed to stubs using self‐adhesive pads and coated with Au via an ion sputtering coater (SBC‐12). SEM images were taken using JSM7610 (Hitachi). Cell counting was performed manually in Image J software (NIH) by using the cell counter function and only enumerating cells that were either clearly rod‐shaped or filamentous.

### Single‐cell sorting

We followed the method described by Diao et al.^83^ for the preparation of *E. coli* cells that express GDGT synthase. The cells were cultured anaerobically at 37°C for 24 h. Post cultivation, we washed the cells thrice with PBS buffer after centrifugation at 4500*g* for 5 min. The cells were then diluted to a density between 10^6^ and 10^7^ cells/ml. For single‐cell morphological sorting, we utilized the Microdroplet Single‐cell Sorting Kit (SCC003101; Qingdao Single‐Cell Biotech. Co., Ltd.). We added 20% of solution B into the cell suspensions to prevent cell adhesion inside the microfluidic channel. We sealed the chip inlet with adhesive tape and subjected the chip to a 10‐min vacuum treatment in a vacuum desiccator. After removal from the vacuum, we immediately inserted the end of the chip's capillary probe into an EP tube filled with cell suspension. This process allowed the micro‐chamber to be filled with the sample. Following this, we used PBS buffer with 20% solution B to flush out residual cells from the chip's channel. We then placed the chip, now its chamber loaded with cells, onto the microscope stage of the EasySort AUTO platform (Qingdao Single‐cell Biotech) for observation. Using the optical tweezer module^83^, ^84^, we captured and transferred the target cells into PCR tubes precisely, ensuring a “What you see is what you get” operation. The sorted cells were numbered, corresponding to the numbered collection tubes. For each selected cell, we added 15 μl of Solution A into its PCR tube to facilitate single‐cell anaerobic culture. Finally, we successfully isolated eight short single cells (1–2 μm) and eight longer single cells (length > 50 μm) for further anaerobic culture and live‐cell morphological studies.

### Live‐cell time‐lapse imaging

For live‐cell time‐lapse imaging, we used the submerged‐sandwich technique for cell growth on gellan gum pads^85^. A gellan gum pads (0.2% w/v) in TM medium were freshly made before experiments. Fresh *E. coli* cells expressing GDGT synthase were collected, and the cell suspension was applied beneath a gel pad on a glass coverslip for bright field microscopy. To maintain an anaerobic condition, 0.5 mM Cys‐HCl was incorporated into the medium, which was then sealed with paraffin wax. The procedure was conducted in triplicate. Microscopic observations were performed using an Eclipse Ti microscope (Nikon) fitted with a 20× objective lens. Subsequent image analysis was carried out employing Image J software (NIH).

### Fluorescence imaging

DAPI (4′, 6‐diamidino‐2‐phenylindole, a fluorescent dye that binds strongly to DNA) (Coolaber, China) was used to observe cells' genetic material. Cells were collected by centrifuge at 6000*g* for 10 min. The cell pellets were subjected to three washes with 1 ml of PBS and then resuspended in 0.1 ml of PBS. Subsequently, 1 μl of DAPI staining solution (100×) was introduced to label the cells. The samples were incubated for 5 min at ambient temperature in a light‐protected environment. Following the labeling process, the cells were centrifuged and washed twice with 1 ml of PBS before being resuspended in PBS to achieve an optimal cell density for imaging purposes. For fluorescence microscopy, 2 μl of the sample was applied to a glass slide at room temperature, atop which a glass coverslip was positioned. Fluorescence images were captured using a Ni‐U fluorescence microscope (Nikon, Japan) with DAPI (Ex: 364 nm; Em: 454 nm) or GFP (Ex: 488 nm; Em: 510 nm).

### Quantitative reverse‐transcription PCR

Total RNA was isolated from *E. coli* cells by first treating the cells with RNAprotect Bacteria Reagent (Qiagen) to stabilize the RNA, followed by RNA extraction using the RNeasy Mini Kit (Qiagen). To eliminate residual DNA, the RNA samples were treated with DNase I (Thermo Fisher Scientific). Subsequently, complementary DNA (cDNA) was synthesized utilizing the iScript cDNA Synthesis Kit (Bio‐Rad). Quantitative PCR (qPCR) was executed with the ChamQ SYBR qPCR Master Mix (Vazyme). The primer pairs utilized for the qRT‐PCR assays are detailed in Table S3. The housekeeping gene *rssA* served as an internal control^86^, and relative expression levels of interested genes were determined using $2^{-\Delta \Delta C_{t}}$ method^87^.

### Intercellular ATP concentration measurement

The concentrations of intercellular ATP were quantified by a luminescence assay kit (Solarbio, China). The GDGT‐producing strain and the wild‐type strain were cultured under anaerobic conditions at 37°C, and harvested at 24, 48, and 72 h incubation time points, and then the ATP concentrations were measured following the manufacturer's protocols.

### Analysis of phyletic patterns

Phyletic pattern analysis was performed for 145 genomes of *Asgardarchaeota* covering all major Asgard lineages^57^, ^58^ and the Asgard archaeal genome IDs were provided in Table S1. For 85 genomes without annotated proteins, we predicted proteins using Prodigal 2.6.3^88^, which was trained on the 12 most complete annotated Asgard genomes. All Asgard archaea proteins were assigned to the Asgard clusters of orthologous genes (asCOGs)^58^ using PSI‐BLAST program^89^. Phyletic patterns for the same enzymes encoded in other archaea were retrieved from the database of arCOGs database^90^, ^91^.

## AUTHOR CONTRIBUTIONS

**Zhirui Zeng:** Funding acquisition (equal); project administration (equal); supervision (lead); writing—original draft (equal). **Huahui Chen:** Data curation (equal); formal analysis (equal); methodology (equal); writing—original draft (equal); writing—review and editing (equal). **Fengfeng Zheng:** Methodology (supporting); resources (supporting); software (supporting); supervision (supporting); writing—review and editing (supporting). **Xi Feng:** Data curation (supporting); investigation (supporting); methodology (supporting); software (supporting); writing—review and editing (supporting). **Zijing Huang:** Methodology (supporting); software (supporting); writing—review and editing (supporting). **Wei Yang:** Data curation (supporting); investigation (supporting); methodology (supporting); writing—review and editing (supporting). **Chuanlun Zhang:** Funding acquisition (supporting); writing—review and editing (supporting). **Wenbin Du:** Methodology (supporting); software (supporting); writing—review and editing (supporting). **Kira S. Makarova:** Methodology (supporting); writing—review and editing (supporting). **Eugene V. Koonin:** Methodology (supporting); writing—review and editing (supporting).

## ETHICS STATEMENT

This study did not involve any experiments on animals or humans.

## CONFLICT OF INTERESTS

The authors declare no conflict of interests.

## Supporting information

Supporting information.

Supporting information.

Supporting information.

## Data Availability

All data are available in the main text, supplementary materials, or the source data file.
