# Supplementary material for: Engineering archaeal membrane‐spanning lipid GDGT biosynthesis in bacteria: Implications for early life membrane transformations
Source: mLife. 2025 Mar 13;4(2):193–204. doi: 10.1002/mlf2.70001 (PMC12042123; doi:10.1002/mlf2.70001)
Supplement: Supplementary file 3 — Supporting information. [file MLF2-4-193-s003.doc]

**SUPPORTING INFORMATION**

**TITLE.** Engineering Archaeal Membrane-Spanning Lipid GDGT Biosynthesis in Bacteria: Implications for Early Life Membrane Transformations

**AUTHORS.** Huahui Chen^1^, Fengfeng Zheng^1^, Xi Feng^1^, Zijing Huang^1^, Wei Yang^1^, Chuanlun Zhang^1^, Wenbin Du^2^, Kira S. Makarova^3^, Eugene V. Koonin^3^, Zhirui Zeng^1,*^

**AFFILIATION.** ^1^Department of Ocean Science and Engineering, Southern University of Science and Technology, Shenzhen 518055, China.

^2^State Key Laboratory of Microbial Resources, Institute of Microbiology, Chinese Academy of Sciences, Beijing 100101, China.

^3^National Center for Biotechnology Information, National Library of Medicine, Bethesda 20894, USA.

***CORRESPONDENT.**

Zhirui Zeng [(zengzr@sustech.edu.cn)](mailto:(zengzr@sustech.edu.cn))


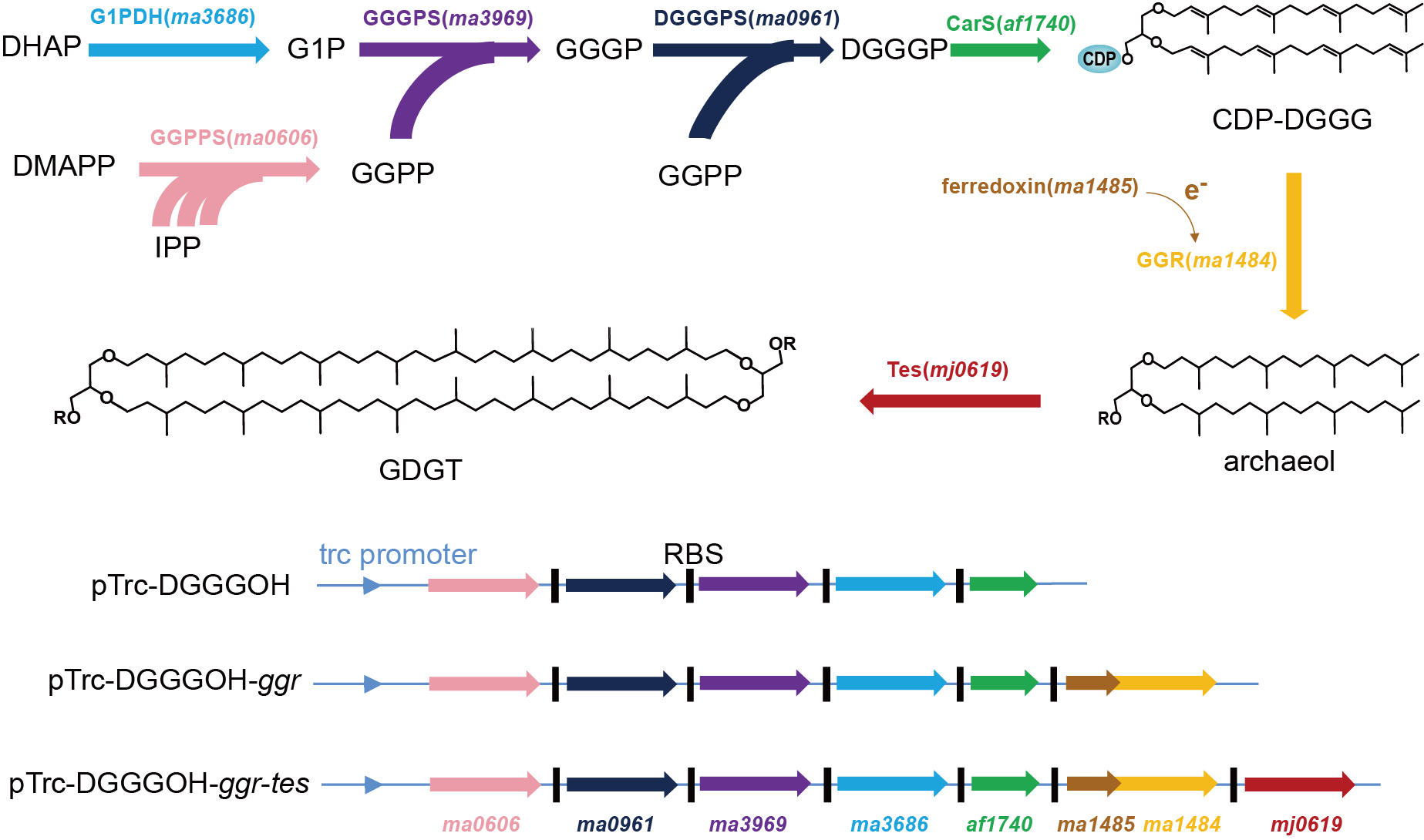


**Figure S1.** The biosynthetic pathway of the archaeal lipids in *E. coli* transformants and the operons in the plasmids used in the study. Abbreviations. DHAP, dihydroxyacetone phosphate; DMAPP, dimethylallyl diphosphate; IPP, isoprenyl diphosphate; G1P, *sn*-glycerol-1-phosphate; GGPP, geranylgeranyl diphosphate; GGGP, geranylgeranylglyceryl phosphate; DGGGP, digeranylgeranylglyceryl phosphate; GGR, geranylgeranyl reductase; Tes, tetraether synthase; GDGT, glycerol dialkyl glycerol tetraether, DGGGOH, digeranylgeranylglycerol.


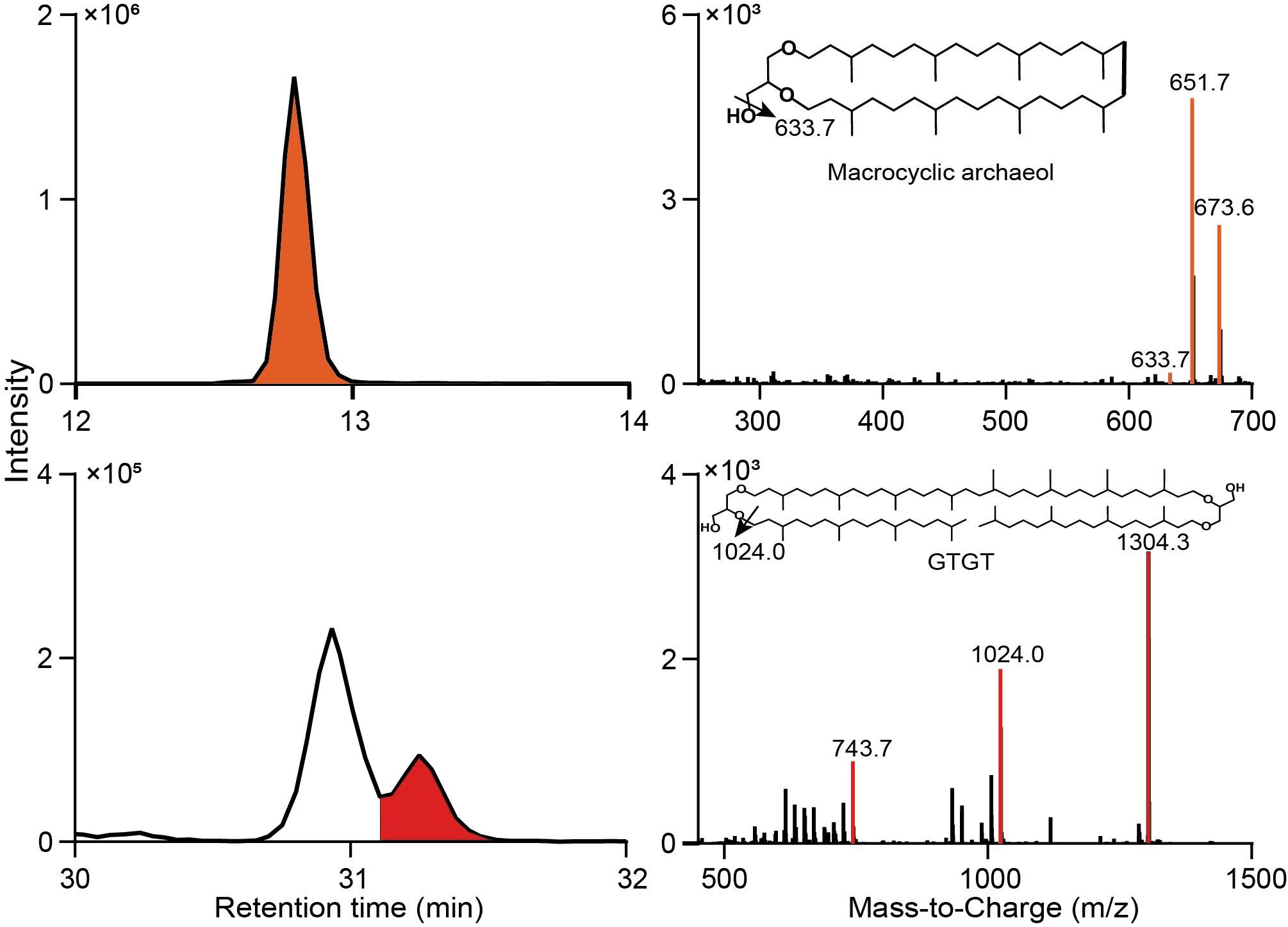


**Figure S2.** Identification of macrocyclic archaeol and GTGT lipids in the GDGT-producing strain of core lipid (CL). Left column shows the LC-MS extracted ion chromatograms (EIC) of macrocyclic archaeol and GTGT compounds extracted from the GDGT-producing strain. Right column shows the MS/MS spectra of corresponding compounds from left column analyzed by RP-LC-HRMS.


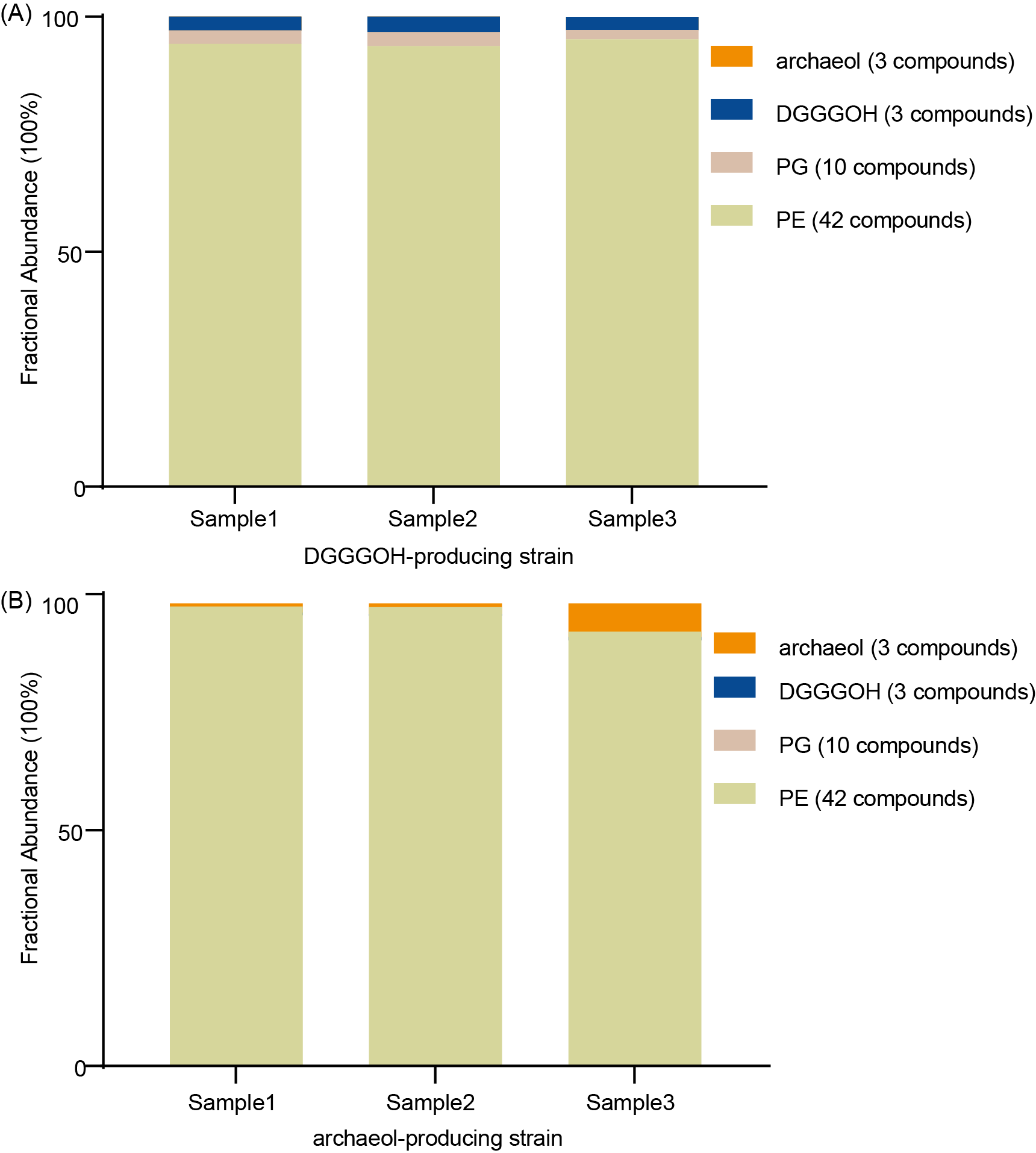


**Figure S3.** IPL-based quantitation of archaeal lipids and bacterial lipids in the DGGGOH-producing strain (A) and the archaeol-producing strain (B). The calculation is based on the results of LC-MS/MS ion intensities analysis (source data file). Data are calculated from three biological replicates. Abbreviations. PG, phosphatidylglycerol; PE, phosphatidylethanolamine.


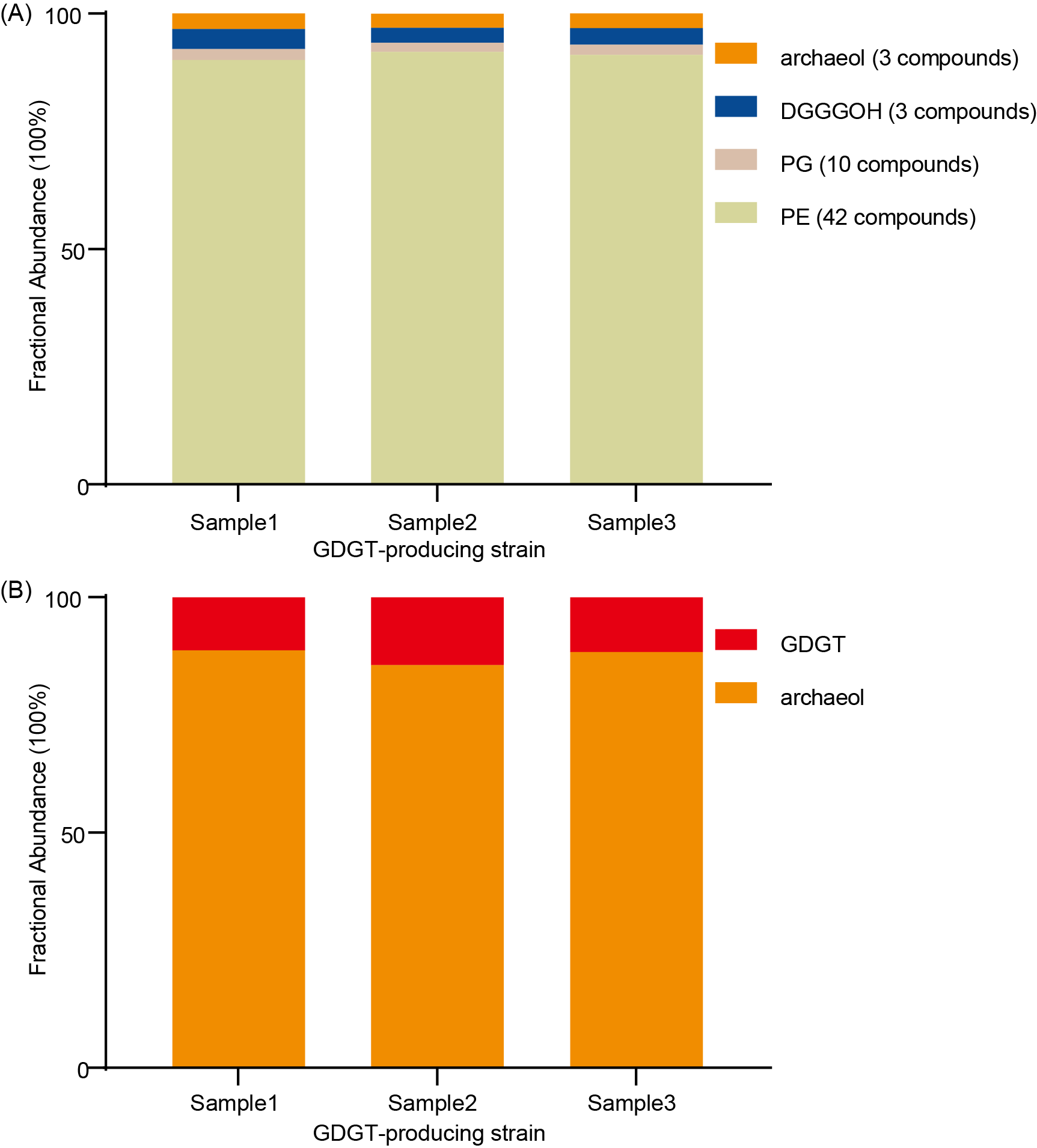


**Figure S4.** The quantification of relative abundance archaeal lipids in GDGT-producing strain. (A) IPL-based quantitation of archaeal lipids and bacterial lipids in GDGT-producing strain. (B) CL-based quantitation of archaeol and GDGT compound in GDGT-producing strain. The calculation is based on the results of LC-MS/MS ion intensities analysis (source data file). Data are calculated from three biological replicates. Abbreviations. PG, phosphatidylglycerol; PE, phosphatidylethanolamine.


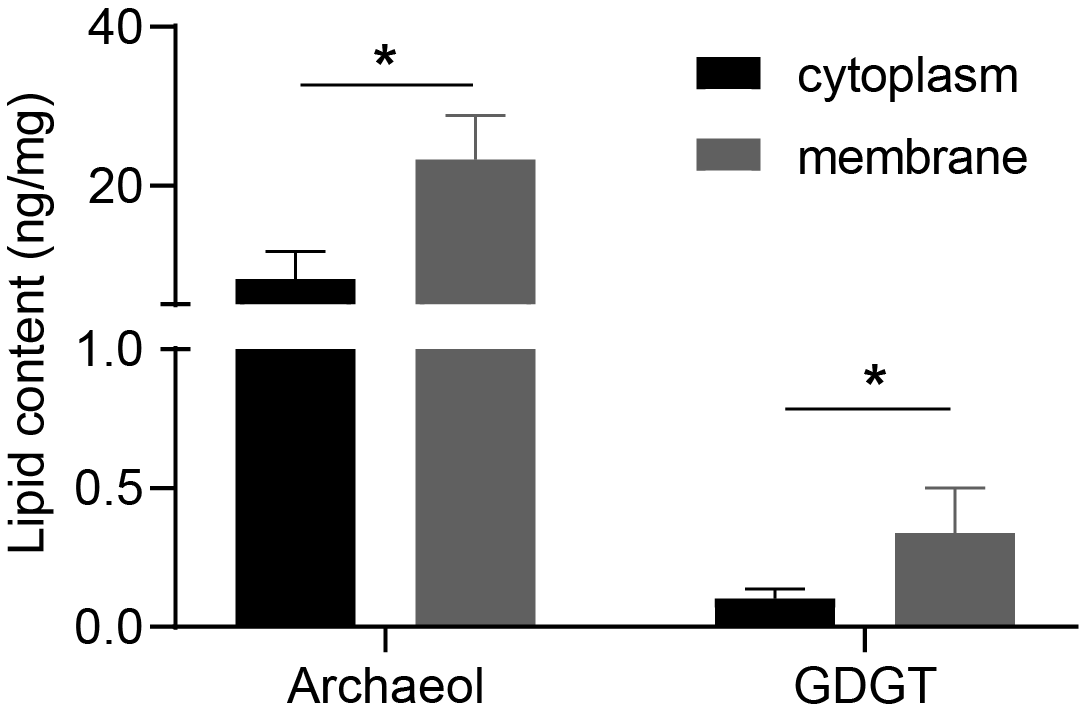


**Figure S5.** The distribution of archaeal lipids in *E. coli* cells. The core lipids are extracted and analyzed from the cell cytosolic and membrane fractions of the GDGT-producing strain respectively. Data are calculated from three biological replicates, and the error bars stand for standard deviation (**p* < 0.05).


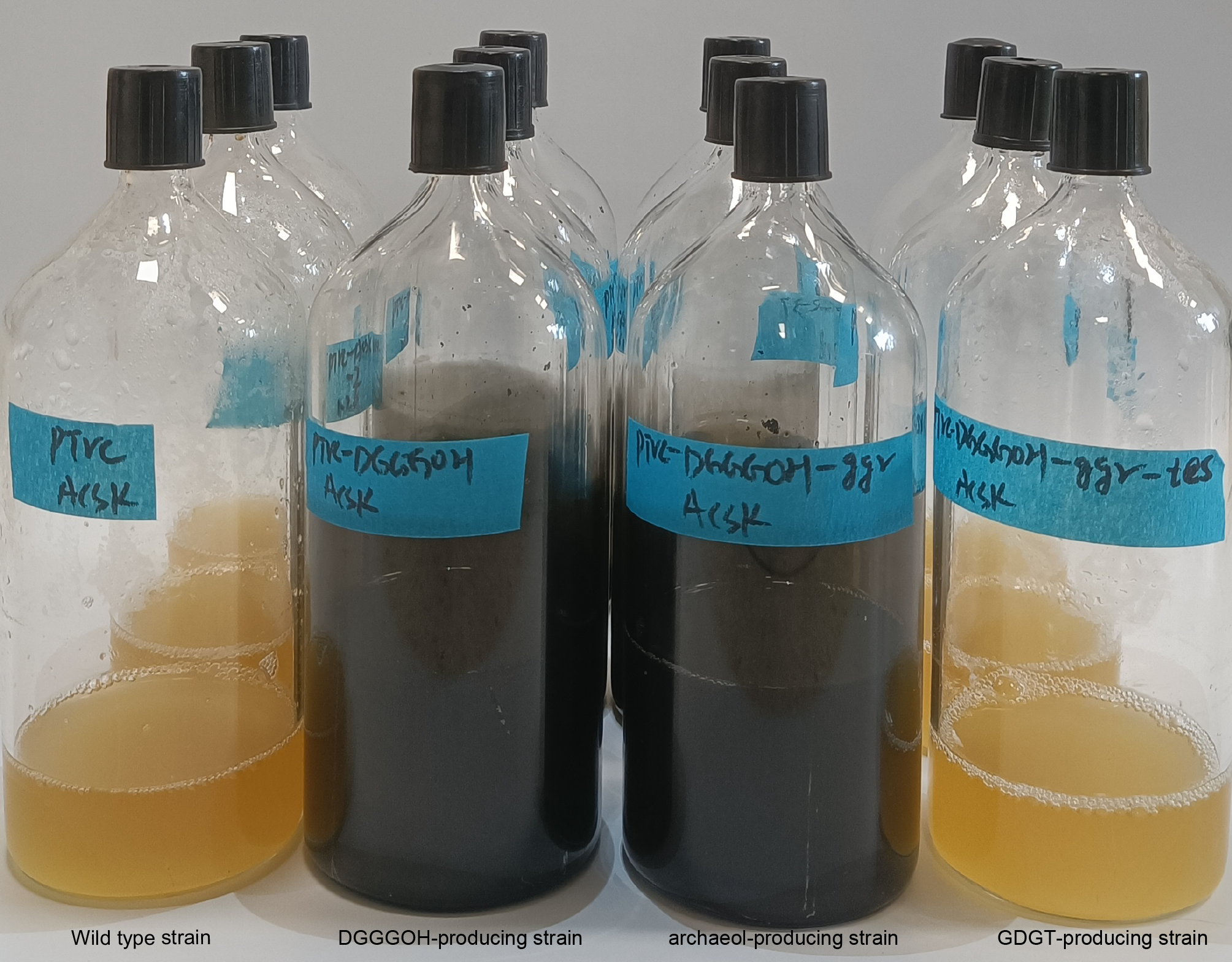


**Figure S6.** The culture color of the wild type, DGGGOH-, archaeol- and GDGT-producing strains.

**
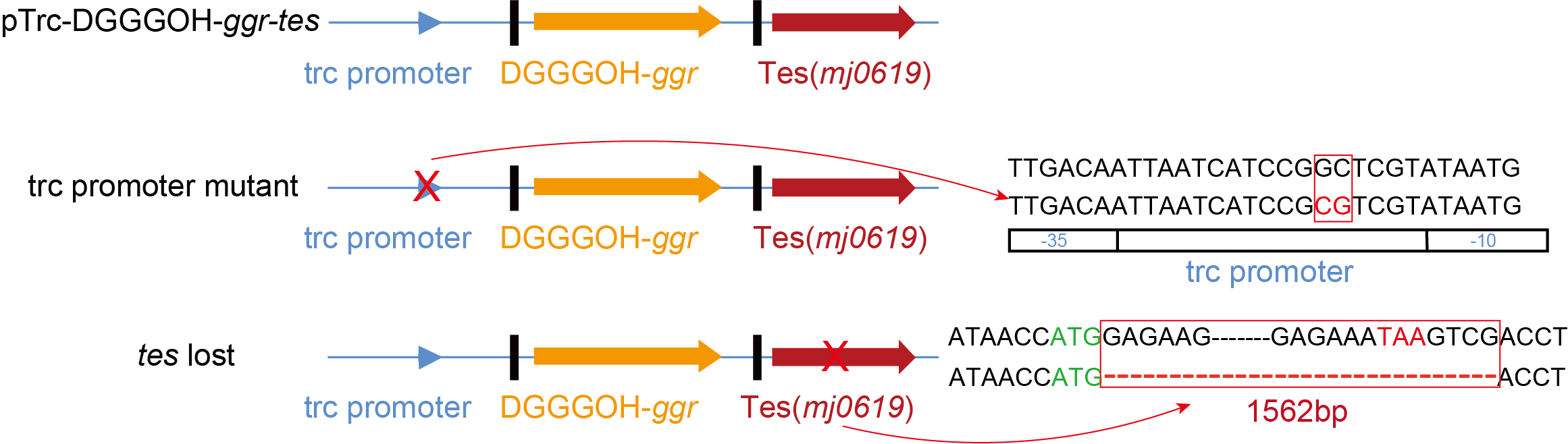
**

**Figure S7.** The mutation occurs on the trc promoter or the *tes* (*mj0619*) gene fragment in the plasmid of pTrc-DGGGOH-*ggr*-*tes* in the GDGT-producing strain.

Table S1. Phyletic patterns of Tes in Asgard archaea metagenome assembled genomes (MAGs).

| **Asgard lineage** | **Assembly ID** | **Genome description** | **Tes** |
| --- | --- | --- | --- |
| *Heimdallarchaeota* | GCA_021513695.1 | Candidatus_Heimdallarchaeum_aukensis_na | 0 |
| *Heimdallarchaeota* | GCA_021513715.1 | Candidatus_Heimdallarchaeum_endolithica_na | 0 |
| *Heimdallarchaeota* | GCA_020348965.1 | Candidatus_Heimdallarchaeota_archaeon_na | 0 |
| *Heimdallarchaeota* | GCA_020351745.1 | Candidatus_Heimdallarchaeota_archaeon_na | 0 |
| *Heimdallarchaeota* | GCA_020353515.1 | Candidatus_Heimdallarchaeota_archaeon_na | 0 |
| *Heimdallarchaeota* | JAEOUF000000000 | YT_re_metabat2_1.002_sub | 1 |
| *Heimdallarchaeota* | JAEOTE000000000 | YT_re_metabat2_1.029_sub | 0 |
| *Heimdallarchaeota* | JAEOTK000000000 | YT_1_bins.1991_sub | 0 |
| *Heimdallarchaeota* | GCA_001940755.1 | Heimdallarchaeacee_AB_125 | 0 |
| *Heimdallarchaeota* | JAHQWT000000000 | Heimdallarchaeaceae_ABR16 | 0 |
| *Heimdallarchaeota* | JAHLEJ000000000 | Heimdallarchaeaceae_GBS11 | 0 |
| *Kariarchaeota* | GCA_002728275.1 | Kariarchaeaceae_RS678 | 0 |
| *Kariarchaeota* | GCA_001940725.1 | Kariarchaeaceae_LC_2 | 1 |
| *Gerdarchaeota* | GCA_011366295.1 | MP5_2_bins.1192 | 0 |
| *Gerdarchaeota* | GCA_011366245.1 | YT_re_metabat2_2.057_sub | 0 |
| *Gerdarchaeota* | GCA_004376455.1 | GCA_004376455.1_ASM437645v1_genomic | 0 |
| *Gerdarchaeota* | GCA_011364945.1 | HSL_bin5.60_strict_reassembled | 0 |
| *Gerdarchaeota* | JAHQWR000000000 | Gerdarchaeales_ABR14 | 0 |
| *Gerdarchaeota* | GCA_003662935.1 | Gerdarchaeales_GBS08 | 0 |
| *Gerdarchaeota* | JAHKKW000000000 | Gerdarchaeales_GBS09 | 0 |
| *Gerdarchaeota* | GCA_003662875.1 | Gerdarchaeales_GBS10 | 0 |
| *Njordarchaeota* | JAGSHR000000000 | Njordarchaeales_A173 | 1 |
| *Njordarchaeota* | JAGSHS000000000 | Njordarchaeales_A3132 | 1 |
| *Njordarchaeota* | JAHKKX000000000 | Njordarchaeales_GBS15 | 0 |
| *Njordarchaeota* | JAHKKY000000000 | Njordarchaeales_GBS16 | 1 |
| *Njordarchaeota* | JAHKKZ000000000 | Njordarchaeales_GBS17 | 1 |
| *Njordarchaeota* | JAHKLA000000000 | Njordarchaeales_GBS18 | 1 |
| *Njordarchaeota* | JAHKLB000000000 | Njordarchaeales_GBS19 | 0 |
| *Njordarchaeota* | JAHKLC000000000 | Njordarchaeales_GBS20 | 1 |
| *Njordarchaeota* | JAHKLD000000000 | Njordarchaeales_GBS21 | 0 |
| *Njordarchaeota* | JAHKLE000000000 | Njordarchaeales_GBS22 | 1 |
| *Njordarchaeota* | JAHKLF000000000 | Njordarchaeales_GBS23 | 1 |
| *Njordarchaeota* | JAHKLG000000000 | Njordarchaeales_GBS24 | 1 |
| *Njordarchaeota* | JAHKLH000000000 | Njordarchaeales_GBS25 | 1 |
| *Njordarchaeota* | JAHKLI000000000 | Njordarchaeales_GBS26 | 1 |
| *Njordarchaeota* | JAGSHQ000000000 | Njordarchaeales_M288 | 1 |
| *Njordarchaeota* | JAHQWF000000000 | Njordarchaeales_TNS08 | 1 |
| *Hodarchaeota* | JAEOTM000000000 | YT_re_metabat2_2.004_sub | 0 |
| *Hodarchaeota* | JAEOSR000000000 | YT_init_bins.019_sub | 0 |
| *Hodarchaeota* | GCA_011364965.1 | HSL_bin2.246_strict_reassembled | 0 |
| *Hodarchaeota* | JAEOUE000000000 | FT_re_metabat2_5.011_sub | 1 |
| *Hodarchaeota* | GCA_003144275.1 | Hodarchaeales_B3_JM_08 | 0 |
| *Hodarchaeota* | GCA_001940645.1 | Hodarchaeales_LC_3 | 1 |
| *Hodarchaeota* | JAHQWW000000000 | Hodarchaeales_WORE3 | 0 |
| *Wukongarchaeota* | JAEORU000000000 | yapct02d125.bin7.59.new | 1 |
| *Wukongarchaeota* | JAEOSI000000000 | yapct02dBC.bin4.70.new | 1 |
| *Sifarchaeota* | JAHQWU000000000 | Sifarchaeia_WORA1 | 1 |
| *Lokiarchaeota* | GCF_008000775.1 | Candidatus_Prometheoarchaeum_syntrophicum_MK-D1 | 1 |
| *Lokiarchaeota* | GCA_025839675.1 | Candidatus_Lokiarchaeum_B-35 | 1 |
| *Lokiarchaeota* | GCA_020343655.1 | Candidatus_Lokiarchaeota_archaeon_na | 1 |
| *Lokiarchaeota* | GCA_020344955.1 | Candidatus_Lokiarchaeota_archaeon_na | 1 |
| *Lokiarchaeota* | GCA_004524425.1 | GCA_004524425.1_ASM452442v1_genomic | 1 |
| *Lokiarchaeota* | GCA_004524515.1 | GCA_004524515.1_ASM452451v1_genomic | 1 |
| *Lokiarchaeota* | JAEOTA000000000 | YT_re_metabat2_1.007_sub | 1 |
| *Lokiarchaeota* | JAEOTR000000000 | YT_re_metabat2_5.013_sub | 1 |
| *Lokiarchaeota* | JAEOTH000000000 | YT_re_metabat2_1.053_sub | 1 |
| *Lokiarchaeota* | GCA_004376705.1 | GCA_004376705.1_ASM437670v1_genomic | 1 |
| *Lokiarchaeota* | GCA_005223125.1 | GCA_005223125.1___Loki_b32 | 1 |
| *Lokiarchaeota* | JAEOTZ000000000 | YT_5_bins.1216_sub | 1 |
| *Lokiarchaeota* | GCA_004524535.1 | GCA_004524535.1_ASM452453v1_genomic | 1 |
| *Lokiarchaeota* | JAEOTQ000000000 | YT_re_metabat2_1.065 | 0 |
| *Lokiarchaeota* | GCA_004375715.1 | GCA_004375715.1_ASM437571v1_genomic | 1 |
| *Lokiarchaeota* | GCA_011364925.1 | DZG_loki_bin1.240_strict_reassembled | 1 |
| *Lokiarchaeota* | GCA_005222975.1 | GCA_005222975.1___Loki_b31 | 1 |
| *Lokiarchaeota* | GCA_011364975.1 | HSL_bin8.338_strict_reassembled | 1 |
| *Lokiarchaeota* | JAEORW000000000 | yapct02d200.bin5.98.new | 1 |
| *Lokiarchaeota* | GCA_004524725.1 | GCA_004524725.1_ASM452472v1_genomic | 1 |
| *Lokiarchaeota* | JAEOSU000000000 | YT_init_bins.039_sub | 1 |
| *Lokiarchaeota* | GCA_004524545.1 | GCA_004524545.1_ASM452454v1_genomic | 1 |
| *Lokiarchaeota* | GCA_001940655.1 | Lokiarchaeales_CR_4 | 1 |
| *Lokiarchaeota* | GCA_000986845.1 | Lokiarchaeales_GC14_75 | 1 |
| *Lokiarchaeota* | JAHQWG000000000 | Lokiarchaeales_ABR01 | 1 |
| *Lokiarchaeota* | JAHQWH000000000 | Lokiarchaeales_ABR02 | 1 |
| *Lokiarchaeota* | JAHQWI000000000 | Lokiarchaeales_ABR03 | 1 |
| *Lokiarchaeota* | JAHQWJ000000000 | Lokiarchaeales_ABR04 | 1 |
| *Lokiarchaeota* | JAHQWK000000000 | Lokiarchaeales_ABR05 | 1 |
| *Lokiarchaeota* | JAHQWL000000000 | Lokiarchaeales_ABR06 | 1 |
| *Lokiarchaeota* | JAHQWM000000000 | Lokiarchaeales_ABR08 | 1 |
| *Lokiarchaeota* | JAHQWP000000000 | Lokiarchaeales_ABR11 | 1 |
| *Lokiarchaeota* | JAHQWQ000000000 | Lokiarchaeales_ABR13 | 1 |
| *Lokiarchaeota* | JAHQWS000000000 | Lokiarchaeales_ABR15 | 1 |
| *Lokiarchaeota* | GCA_003662865.1 | Lokiarchaeales_GBS14 | 1 |
| *Lokiarchaeota* | JAHQWX000000000 | Lokiarchaeales_WORB4 | 1 |
| *Lokiarchaeota* | JAHQWY000000000 | Lokiarchaeales_WORC5 | 1 |
| *Helarchaeota* | JAEOSY000000000 | YT_metabat2_results_m2000.4001_refined_after_  dastool | 1 |
| *Helarchaeota* | GCA_011365055.1 | HSL_bin10.384_bin.1.complete | 1 |
| *Helarchaeota* | GCA_005191415.1 | Helarchaeales_HELGBA | 1 |
| *Helarchaeota* | GCA_005191425.1 | Helarchaeales_HELGBB | 1 |
| *Thorarchaeota* | GCA_020348985.1 | Candidatus_Thorarchaeota_archaeon_na | 1 |
| *Thorarchaeota* | GCA_020355105.1 | Candidatus_Thorarchaeota_archaeon_na | 1 |
| *Thorarchaeota* | GCA_002825515.1 | Maipo_11 | 1 |
| *Thorarchaeota* | JAEORX000000000 | yapct02d200.bin7.15.new | 1 |
| *Thorarchaeota* | GCA_004376265.1 | GCA_004376265.1_ASM437626v1_genomic | 1 |
| *Thorarchaeota* | GCA_002825465.1 | Maipo_8 | 1 |
| *Thorarchaeota* | GCA_002825535.1 | Maipo_9 | 1 |
| *Thorarchaeota* | GCA_004524565.1 | GCA_004524565.1_ASM452456v1_genomic | 1 |
| *Thorarchaeota* | JAEOSL000000000 | yapct02d500.bin9.44.new_sub | 1 |
| *Thorarchaeota* | JAEOUN000000000 | FT_re_metabat2_5.026 | 1 |
| *Thorarchaeota* | GCA_004524595.1 | GCA_004524595.1_ASM452459v1_genomic | 1 |
| *Thorarchaeota* | JAEOUM000000000 | FT_re_metabat2_5.021_sub | 1 |
| *Thorarchaeota* | JAEOUG000000000 | FT_re_metabat2_1.004 | 1 |
| *Thorarchaeota* | JAEORS000000000 | yapct02d125.bin7.129.new | 1 |
| *Thorarchaeota* | JAEORN00000000 | MP5_init_bins.2226_sub | 0 |
| *Thorarchaeota* | GCA_004524445.1 | GCA_004524445.1_ASM452444v1_genomic | 1 |
| *Thorarchaeota* | GCA_004524435.1 | GCA_004524435.1_ASM452443v1_genomic | 1 |
| *Thorarchaeota* | GCA_001940705.1 | Thorarchaeia_AB_25 | 0 |
| *Thorarchaeota* | GCA_003345545.1 | Thorarchaeia_GBS30 | 1 |
| *Thorarchaeota* | GCA_003345595.1 | Thorarchaeia_GBS31 | 1 |
| *Thorarchaeota* | GCA_003345555.1 | Thorarchaeia_GBS32 | 0 |
| *Thorarchaeota* | GCA_001563335.1 | Thorarchaeia_SMTZ1_45 | 1 |
| *Thorarchaeota* | GCA_001563465.1 | Thorarchaeia_SMTZ_45 | 0 |
| *Thorarchaeota* | GCA_001563325.1 | Thorarchaeia_SMTZ_83 | 0 |
| *Thorarchaeota* | JAGSHN000000000 | Thorarchaeia_A361 | 1 |
| *Thorarchaeota* | JAGSHO000000000 | Thorarchaeia_A381 | 1 |
| *Thorarchaeota* | JAGSHP000000000 | Thorarchaeia_A399 | 1 |
| *Thorarchaeota* | JAHQWN000000000 | Thorarchaeia_ABR09 | 0 |
| *Thorarchaeota* | JAHQWO000000000 | Thorarchaeia_ABR10 | 1 |
| *Thorarchaeota* | GCA_003662815.1 | Thorarchaeia_GBS28 | 1 |
| *Thorarchaeota* | GCA_003662805.1 | Thorarchaeia_GBS29 | 1 |
| *Thorarchaeota* | GCA_003662775.1 | Thorarchaeia_GBS33 | 1 |
| *Thorarchaeota* | GCA_003662765.1 | Thorarchaeia_GBS34 | 1 |
| *Thorarchaeota* | JAHQWZ000000000 | Thorarchaeia_WORH6 | 1 |
| *Hermodarchaeota* | JAEOSV000000000 | YT_init_bins.046 | 1 |
| *Hermodarchaeota* | JAEOUH000000000 | FT_re_metabat2_1.005_sub | 1 |
| *Hermodarchaeota* | JAEOSK000000000 | yapct02dBC.bin9.105.new | 1 |
| *Hermodarchaeota* | JAEOTO000000000 | YT_re_metabat2_2.032_sub | 1 |
| *Hermodarchaeota* | JAEOTB000000000 | YT_re_metabat2_1.014_sub | 0 |
| *Hermodarchaeota* | JAHQWV000000000 | Hermodarchaeia_WORB2 | 1 |
| *Baldrarchaeota* | JAEOSG000000000 | yapct02d30.bin4.67.new | 1 |
| *Baldrarchaeota* | JAEOSH000000000 | yapct02d30.bin9.72.new | 1 |
| *Baldrarchaeota* | JAHKLJ000000000 | Baldrarchaeia_GBS02 | 1 |
| *Baldrarchaeota* | JAHKLK000000000 | Baldrarchaeia_GBS03 | 1 |
| *Baldrarchaeota* | JAHKLL000000000 | Baldrarchaeia_GBS04 | 1 |
| *Odinarchaeota* | GCA_001940665.2 | Candidatus_Odinarchaeum_yellowstonii | 1 |
| *Odinarchaeota* | JAHQXC000000000 | Odinarchaeia_RPA3 | 1 |
| *Jordarchaeota* | JAHKKT000000000 | Jordarchaeia_GBS05 | 1 |
| *Jordarchaeota* | JAHKKU000000000 | Jordarchaeia_GBS06 | 1 |
| *Jordarchaeota* | JAHKKV000000000 | Jordarchaeia_GBS07 | 1 |
| *Jordarchaeota* | JAHAWQ000000000 | Jordarchaeia_JZB50 | 1 |
| *Jordarchaeota* | JAHAWR000000000 | Jordarchaeia_QC4B49 | 1 |
| *Jordarchaeota* | JAHAWS000000000 | Jordarchaeia_QZMA23B3 | 1 |
| *Jordarchaeota* | JAHAWT000000000 | Jordarchaeia_QZMA2B5 | 1 |
| *Jordarchaeota* | JAHAWU000000000 | Jordarchaeia_QZMA3B5 | 1 |
| *Jordarchaeota* | JAHQXA000000000 | Jordarchaeia_RPD1 | 1 |
| *Jordarchaeota* | JAHQXB000000000 | Jordarchaeia_RPF2 | 1 |

**Table S2.** Strains used in this study.

| **Strains** | **Genotype** | **Source or reference** |
| --- | --- | --- |
| *Escherichia coli* DH10B | F^−^ *endA1 recA1 galE15 galK16 nup GrpsL ΔlacX74 Φ80lacZΔM15 araD139 Δ(ara,leu)7697 mcrA Δ(mrr-hsdRMS*-*mcrBC)λ^−^* | Paula V. Welander  (Stanford University) |

**Table S3.** Plasmids used in this study.

| Plasmids | Description | Source or reference |
| --- | --- | --- |
| pTrc | *E. coli* expression plasmids containing pBR322 ori, lacUV5 promoter, Amp^R^ | (1) |
| pTrc-DGGGOH | Five archaeal lipids biosynthase gene (*ma0606*, *ma0961*, *ma3969*, *ma3686* from *M. acetivorans*, *af1740* from *Archaeoglobus fulgidus*) were amplified by PCR with primers DGGGOH1-5 F/R respectively and cloned into the NcoI and XbaI site of pTrc plasmid. | This work |
| pTrc-DGGGOH-*ggr* | The fragment *ggr* (*ma1484*-*ma1485* from *M. acetivorans*) was amplified by PCR with primers ggrF/R and cloned into the XbaI site of pTrc-DGGGOH plasmid. | This work |
| pTrc-DGGGOH-*ggr*-*tes* | *Methanocaldococcus jannaschii* *mj0619* was amplified by PCR with primers tesF/R and cloned into the SalI site of pTrc-DGGGOH-*ggr* plasmid. | This work |
| pSRK | pBBR1 ori, lacUV5 promoter, Gm^R^ | (2) |
| pSRK-*ftsZ*-*sfGFP* | The fragment of *ftsZ* and *sfGFP* were amplified by PCR with primers P3F/R and P4F/R and cloned into the NdeI and HindIII site of pSRK plasmid. | This work |
| pJBEI2997 | p15A ori, LacUV5promoter, Cm^R^ | (3) |
| pDB1281 | Contain the *Azotobacter vinelandii* isc (iron sulfur cluster) operon under the control of an arabinose-inducible promoter, Kan^R^ | (4) |
| pBAD42-BtuCEDFB | Contain *E. coli* K12 *btuC*,*btuE*,*btuD*,*btuF* and *btuB* gene under the control of an pBAD promoter, Spec^R^ | (5) |

**Table S4.** Primers used in this study.

| Primer | Sequence(5’-3’) | Note |
| --- | --- | --- |
| For plasmid construction | | |
| P1F | CAATTAATCATCCGGCTCGT | pTrc plasmid check |
| P1R | CGCTTCTGCGTTCTGATTTA |
| DGGGOH1F | ATAACAATTTCACACAGGAAACAGACCATGCTTATGATGCTTATTGATGAG | *ma0606* cloning into pTrc plasmid |
| DGGGOH1R | GGTTTTTTCCTCCTATTCTCAGTATTCCCTTGCAATCATA |
| DGGGOH2F | TATATGATTGCAAGGGAATACTGAGAATAGGAGGAAAAAACCATGTCTGCCGGAATACG | *ma0961* cloning into pTrc plasmid |
| DGGGOH2R | CCTGCATTTTAGTTTCCTCCTACGTCATACACCGGCAATGA |
| DGGGOH3F | GGTGTATGACGTAGGAGGAAACTAAAATGCAGGTGGAAGCACA | *ma3969* cloning into pTrc plasmid |
| DGGGOH3R | AATTTCATGTTACTTTCCTCCTTATTTAGCTTTTTATAGCTGATACGATCTC |
| DGGGOH4F | AAAGCTAAATAAGGAGGAAAGTAACATGAAATTGACCATCAATAAAA | *ma3686* cloning into pTrc plasmid |
| DGGGOH4R | TCCTCCTCCTAGGATCCCCGGGTACTCAACTTATGACCTTTGTGATT |
| DGGGOH5F | GTCATAAGTTGAGTACCCGGGGATCCTAGGAGGAGGAAACTAAAATGCTGGATCTGA | *af1740* cloning into pTrc plasmid |
| DGGGOH5R | ATGCCACACCATCTTTAGTTACCTCCTAGGTTACCACGGCACTTCTTTCAGGTTCAG |
| ggrF | CCTGAAAGAAGTGCCGTGGTAACCTAGGAGGTAACTAAAGATGGTGTGGCATTATACTAATGATGT | *ggr* (*ma1484* and *ma1485*) cloning into pTrc-DGGGOH plasmid |
| ggrR | AAGCTTGCATGCCTGCAGGTCGACTTTATGTCCTGTTCTTCATCACTTCTTTTG |
| tesF | AGAACAGGACATAAAGAGGAGGAAATAACCATGGAGAAGAAAACCCTGAGCCT | *tes* (*mj0619*) cloning into pTrc-DGGGOH-*ggr* plasmid |
| tesR | CTTGCATGCCTGCAGGTCGACTTATTTCTCAAATTGCGGGTGGCT |
| P2F | CAGTGAGCGCAACGCAATTAA | pSRK plasmid check |
| P2R | CCGGTAAACCAGCAATAGACA |
| P3F | TCACACAGGAGGCAAGCATATGTTTGAACCAATGGAACTTACCAATGAC | *ftsZ* and *sfGFP* cloning into pSRK plasmid to construct pSRK-*ftsZ*-*sfGFP* |
| P3R | CTTTACGCATCGCGCTATCAGCTTGCTTACGCAGGAATG |
| P4F | AGCTGATAGCGCGATGCGTAAAGGTGAAGAACTGTTCAC |
| P4R | GTCGACGGTATCGATAAGCTTTTATTTGTAGAGCTCATCCATGCCGT |
| P5F | CGTCCGGCGTAGAGGATC | pJBEI2997 plasmid check |
| P5R | TGCAGGCCCGCTAATTGC |
| P6F | GCTCAAGGACTCCTTCGAG | pDB1281 plasmid check |
| P6R | CAGTCAATCGGGGGGGTTGAAAAG |
| P7F | AAGCTTCGCTGCTGACGG | pBAD42-BtuCEDFB plasmid check |
| P7R | GATTTAATCTGTATCAGG |
|  |  |  |
| For RT-qPCR | | |
| recA-qRT-F | CAGGGCGTCACAGATTTCCA |  |
| recA-qRT-R | ACCACGCTGACGCTGC |  |
| lexA-qRT-F | AATTGTTTCCGGCGCATCAC |  |
| lexA-qRT-R | GCAGGAAATCAGCATTCGGC |  |
| sulA-qRT-F | CCCAGATGCCTGAACCCATT |  |
| sulA-qRT-R | ATCAGCCCATGATGACGCAA |  |
| lon-qRT-F | GAAAACGAAGCCCTGAAGCG |  |
| lon-qRT-R | GGCACCTGTACCATCCAGTC |  |
| ftsZ-qRT-F | ATCCCGAACGACAAACTGCT |  |
| ftsZ-qRT-R | ATACCTTGCACAGCGCCTTT |  |
| rssA-qRT-F | ACGCCTGATGGATCTCTCCT |  |
| rssA-qRT-R | TGCGCGAATAGCAAGATGGA |  |

**Movie S1**

The live-cell time-lapse microscopy imaging illustrates the elongation process of the GDGT-producing strain ending with explosive cell lysis. Relates to Fig. 2**A**.

**References**

1. Amann E, Ochs B, Abel KJ. Tightly regulated tac promoter vectors useful for the expression of unfused and fused proteins in *Escherichia coli*. *Gene*. 1988;**69**:301–315.

2. Banta AB, Wei JH, Gill CCC, Giner JL, Welander PV. Synthesis of arborane triterpenols by a bacterial oxidosqualene cyclase. *Proc Natl Acad Sci U S A.* 2017,**114**:245–250.

3. Peralta-Yahya PP, Ouellet M, Chan R, Mukhopadhyay A, Keasling JD, Lee TS. Identification and microbial production of a terpene-based advanced biofuel. *Nat Commun.* 2011;2:483.

4. Zheng L, Cash VL, Flint DH, Dean DR. Assembly of iron-sulfur clusters. Identification of an iscSUA-hscBA-fdx gene cluster from *Azotobacter vinelandii*. *J Biol Chem.* 1998;**273**;13264–13272.

5. Lanz ND, Blaszczyk AJ, McCarthy EL, Wang B, Wang RX, Jones BS, Booker SJ. Enhanced solubilization of class B radical S-Adenosylmethionine Methylases by improved cobalamin uptake in *Escherichia coli*. *Biochemistry.* 2018;**57**:1475–1490.
